# Supplementary material for: A high-resolution mRNA expression time course of embryonic development in zebrafish
Source: eLife. 2017 Nov 16;6:e30860. doi: 10.7554/eLife.30860 (PMC5690287; doi:10.7554/eLife.30860)
Supplement: Supplementary file 6. [file elife-30860-supp6.zip › biolayout-clusters-files/Cluster128-genes.html]

Cluster128


# Cluster128: Genes

| Ensembl ID | Gene Name | Chr | Start | End | Biotype |
| --- | --- | --- | --- | --- | --- |
| ENSDARG00000104429 | ENSDARG00000104429 | 25 | 88622 | 95743 | protein\_coding |
| ENSDARG00000057652 | dbpb | 16 | 13709573 | 13723355 | protein\_coding |
| ENSDARG00000098831 | gcdhb | 3 | 13787580 | 13806538 | protein\_coding |
| ENSDARG00000101239 | hsd17b4 | 8 | 190535 | 214498 | protein\_coding |
| ENSDARG00000062096 | pm20d1.2 | 23 | 18067448 | 18091603 | protein\_coding |
| ENSDARG00000057231 | rorca | 8 | 18854691 | 18869010 | protein\_coding |
| ENSDARG00000104347 | rtn4b | 13 | 129918 | 139991 | protein\_coding |
| ENSDARG00000098121 | si:ch73-14h10.4 | 6 | 28232428 | 28234403 | lincRNA |
| ENSDARG00000103983 | slc30a10 | 13 | 51757317 | 51764306 | protein\_coding |
| ENSDARG00000006266 | stat1a | 22 | 12773792 | 12802185 | protein\_coding |
